# Fascial Nomenclature: Update 2022

**DOI:** 10.7759/cureus.25904

**Published:** 2022-06-13

**Authors:** Bruno Bordoni, Allan R Escher, Filippo Tobbi, Luigi Pianese, Antonio Ciardo, Jay Yamahata, Saul Hernandez, Oscar Sanchez

**Affiliations:** 1 Physical Medicine and Rehabilitation, Foundation Don Carlo Gnocchi, Milan, ITA; 2 Anesthesiology and Pain Medicine, H. Lee Moffitt Cancer Center and Research Institute, Tampa, USA; 3 Osteopathy, Poliambulatorio Medico e Odontoiatrico, Varese, ITA; 4 Physical Medicine and Rehabilitation, 3C+A Health and Rehabilitation, Roma, ITA; 5 Osteopathy, Grupo Thuban - Fundación Europea Medicina Tradicional Complementaria e Integrativa (FMTCI) - Universidad Europea del Atlántico (UNEATLANTICO), Madrid, ESP; 6 Osteopathy, Palms College of Osteopathic Medicine, Osaka, JPN; 7 Osteopathy, La Escuela Nacional Mexicana de Medicina Osteopática, México, MEX; 8 Physical Therapy, Clínica Sanares, Guadalajara, ESP

**Keywords:** senoma, glymphatic, pain, manual therapy, holography fascia, osteopathy, osteopathic, myofascial, fascintegrity, fascia

## Abstract

The connective tissue or fascia plays key roles in maintaining bodily function and health. The fascia is made up of solid and fluid portions, which interpenetrate and interact with each other, forming a polymorphic three-dimensional network. In the vast panorama of literature there is no univocal thought on the nomenclature and terminology that best represents the concept of fascia. The Foundation of Osteopathic Research and Clinical Endorsement (FORCE) organization brings together various scientific figures in a multidisciplinary perspective. FORCE tries to find a common nomenclature that can be shared, starting from the scientific notions currently available. Knowledge of the fascial continuum should always be at the service of the clinician and never become an exclusive for the presence of copyright, or commodified for the gain of a few. FORCE is a non-profit organization serving all professionals who deal with patient health. The article reviews the concepts of fascia, including some science subjects rarely considered, to gain an understanding of the broader fascial topic, and proposing new concepts, such as the holographic fascia.

## Introduction and background

The fascial tissue in the collective imagination is associated with a solid structure, which can lead to problems related to pain or a disturbance of motor functions. Despite the easy connection between solid fascia and functional or symptomatic behaviors that can arise from this tissue, when we try to investigate its real connections and/or functions, we do not have precise information. To give some examples, the iliotibial band (ITB) is connected to muscles such as the gluteus maximus and the tensor fascia latae; an alteration of the structure and function of the ITB causes pain and a disturbed distribution of the mechanical tensions suffered by the knee area [1]. Despite the anatomy describing where it is located and the histology describing the morphological structure, we do not know in detail how the exchanges of biomechanical information take place between the adjacent myofascial structures and the ITB, similarly as we do not understand why this area behaves in different ways in different subjects [1,2]. There is not always agreement on the definition or description or function of the different fascial areas. The precaecocolic fascia or Jackson's membrane is described in different ways (long or short, thick or thin, translucent or opaque, membrane or fascia), depending on the anatomical subjectivity, the function of which is not always known [3]. The value of the fascia changes according to the health professional. For the surgeon, the endopelvic fascia is a structure that plays an important role in the function and support of the viscera, while for the anatomist it is a weak layer consisting of areolar tissue with the function of covering the pelvic viscera [4].

With the advancement of technology, more detailed structures are observed, naming a certain tissue with new terminologies or replacing the previous ones. The circumneurium replaces the previous name of paraneurium or paraneural sheath, which is a non-neural tissue or fascia that covers most nerves and is more external than the underlying layer or epineurium, divided into an external and an internal border; the epineurium may contain in its thickness (internal and external) adipocyte-containing compartments, which may be absent or present depending on the overall thickness of the nerve [5]. We do not know in detail the functions of this circumneurium with respect to the biomechanics of the nerve and with respect to the surrounding tissues [6]. With the improvement of surgical techniques that go hand-in-hand with research, descriptions of different fascial relationships and anatomical continuities increase. The superficial muscular aponeurotic system (SMAS) is an important anatomical area for facial plastic surgery, which connects the superficial area of the upper lip, the nasolabial fold, the frontal, parotid, zygomatic and infraorbital portion, part of the platysma muscle, and the sternocleidomastoid muscle, creating a complicated fascial network [7]. The surgeon must consider these connections before organizing the surgery.

Fascial continuity can be a source of pain, in chronic or acute pathological conditions; manual treatment aimed at the fascial system can relieve associated symptoms. In patients with hemophilic elbow arthropathy, it is evident that a manual treatment improves the symptomatological picture and the quality of life; the perception of sternal pain decreases, with respiratory functional improvements, in patients undergoing median sternotomy for cardiac surgery through manual fascial treatments [8,9]. It is important for the doctor to know exactly where to apply subcutaneous drug therapy. In the case of greater trochanteric pain syndrome, the use of an ultrasound (ultrasound-guided fascial plane block) becomes fundamental to discern the depth and location of the fascial anatomical structures to insert the needle correctly; the same evaluation approach (fascial ultrasound) can be used for other anesthesia procedures for surgery [10,11]. The fascial tissue can present structural alterations, such as ossifications, without apparent functional disturbances or pain, or a fascial variant not previously recorded in the literature may be highlighted, without necessarily knowing its function [12,13]. The article reviews the historical evolution of the fascia, the evolution of some theoretical models to understand the fascia, and takes a look at some scientific subjects not always taken into consideration by the multiplicity of publications.

## Review

Historical evolution of the fascia

The first to understand that the fascia is a complex system from an anatomical point of view were the ancient Egyptians (2,500 BC); the fascia is a Latinization of the Greek word "taenia" or "ταινία" (ribbon/band); the Romans gave the plural "fasciae" and singular "fascia" [14]. In 1615, Crooke used the term fascia as an anatomical structure, later followed by other authors, in particular, to identify a membrane, a structure that connects and supports. In the 1700s, different terms began to be used to indicate something membranous, aponeurotic and tendon, especially related to the anatomy of skeletal muscles [14]. In 1780, Dr. Simmons began to understand that fascia or connective tissue involved a large part of the body, with connected it and covered vessels, nerves, and organs [14]. In the first half of the 1800s, various names began to be given to the fascia, based on its location, function, and shape, especially inherent to the musculature. In 1851, Dr. Wilson began talking about layers, defining the fascia starting from the dermis (under the epidermis) [14]; the concept can be found in Gray's 1858 anatomy book [15]. In the twentieth century, after many publications were using the term fascia, some anatomical clarifications of the terminology came out by groups of anatomists and researchers, such as the International Committee for Anatomical Nomenclature (1983) and the Federative Committee of Anatomical Terminology (1998). These last two groups highlighted some words such as "fascia superficialis" and "fascia profunda", comparing the fascial tissue as "sheaths, sheets, or other aggregations of dissectable connective tissue" [14]. The name was given on the basis of the related tissue and the depth of the tissue layers (visceral fascia, fascial planes, fascial system, investing fascia); Towards the end of the 1900s, discussions began on the fact that fascia is a connective tissue in continuity with all other connective tissues (without beginning and without end) [14]. In the twenty-first century, the Fascia Research Congress (FRC) (2007) began to consider some new structures belonging to the fascia, such as joint capsules [14]. From the 2014 FRC, a group of experts was born, the Fascia Nomenclature Committee (FNC), which in 2019 reaffirmed its concept of fascia: "a fascia is a sheath, a sheet, or any other dissectible aggregations of connective tissue that forms beneath the skin to attach, enclose, and separate muscles and other internal organs" [16]. For the FNC, the concept of the fascial system is contained in the following definition:

“consists of the three-dimensional continuum of soft, collagen-containing, loose and dense fibrous connective tissues that permeate the body. It incorporates elements such as adipose tissue, adventitiae and neurovascular sheaths, aponeuroses, deep and superficial fasciae, epineurium, joint capsules, ligaments, membranes, meninges, myofascial expansions, periostea, retinacula, septa, tendons, visceral fasciae, and all the intramuscular and intermuscular connective tissues including endo-/peri-/epimysium. The fascial system surrounds, interweaves between, and interpenetrates all organs, muscles, bones and nerve fibers, endowing the body with a functional structure, and providing an environment that enables all body systems to operate in an integrated manner” [17].

If we take a look at the literature that appears on PubMed, the first text that mentions the fascia in the medical and clinical field is dated 1814, while the term "fasciæ" appears in a journal of 1824 [18,19]. We can read the term "myofascial" for the first time in an article from 1952 [20]. Only in 1991 were the words “fascial system” used to try to describe the fascial continuum [21]. If we carefully look at the literature on PubMed, other terms have been used to define and give an idea of fascial continuity, before the more familiar current terms (fascial system): superficial musculoaponeurotic system; connective tissue system; fibroelastic network; fascial plane system; myoelastic system; musculoperiosteal system and fascioperiosteal system; elastic system; and fasciocutaneous system [22-30]. In 2019, the Federative International Program for Anatomical Terminology (FIPAT), the organization that includes anatomists (International Federation of Associations of Anatomists (IFAA)), gave an update on the concept of fascia:

“Fasciae/fascia of muscles (deep fascia); investing fascia; fascia of individual muscle (fascia sheath); intermuscular septum; compartment; retinaculum (fasciae of body cavities); parietal fascia; visceral fascia/fasciae; extraperitoneal fascia (extraserosal fascia); extraperitoneal ligament; superficial/deep, middle layer/investing; aponeurosis; membrane; ligament; visceral ligament; tendinous; ring; canal; hiatus; triangle; fat” [31].

Foundation of Osteopathic Research and Clinical Endorsement (FORCE)

**Figure 1:**
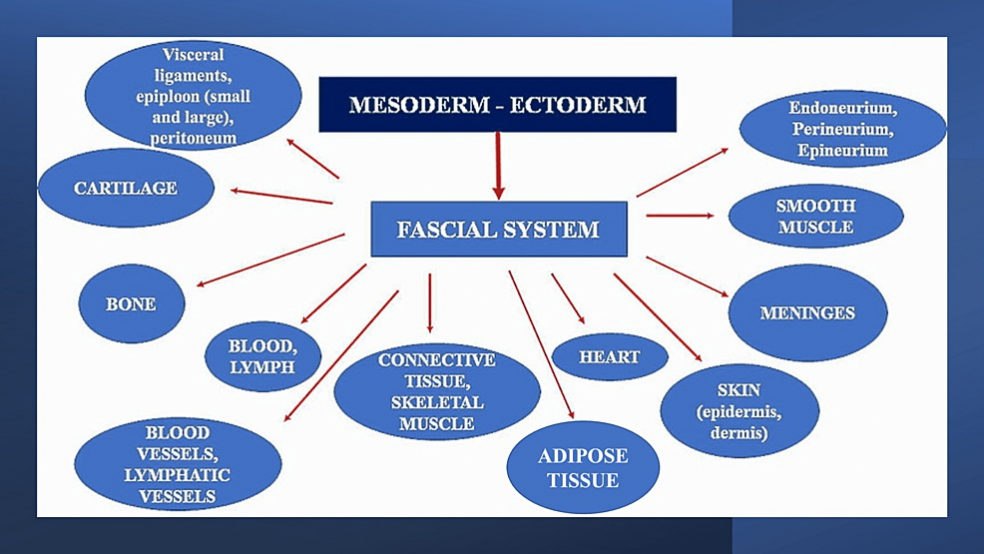
The figure illustrates the tissues that could be considered as fascial tissue, originating from the mesodermal and ectodermal sheets Reprinted with permission from the authors' earlier article; Bordoni et al. [32]

What emerges from all these definitions and terminologies is that the fascia and its continuity are considered only as solid tissue from an anatomical, histological, and topographical perspective. In the breadth of scientific literature, we can find another (non-profit) organization, namely, the FORCE; the latter includes various health professionals, from the surgeon to the bioengineer, from the osteopathic physician to the chiropractor, from the physiotherapist to the clinical doctor [16]. FORCE was founded in 2013, pursuing a functional footprint and looking at all scientific subjects, as to understand a tissue or a body cell, all scientific disciplines can help to understand its different functions [32]. FORCE is in line with the medical nomenclature, considering not only blood and lymph as connective tissue but also bones [16,32,33]. The term fascial continuum has appeared in our articles since 2014, taking its cue from a 1984 text [34,35]. FORCE starts from embryological knowledge, from which it is possible, in a second analysis, to define a tissue; from this fundamental passage, we can affirm that different tissues that derive from the mesoderm and the ectoderm are connective-fascial tissues [16,32,36-44]. FORCE defines the fascial continuum (fascial system) as follows:

“the fascia is any tissue that contains features capable of responding to mechanical stimuli. The fascial continuum is the result of the evolution of the perfect synergy among different tissues, liquids, and solids, capable of supporting, dividing, penetrating, feeding, and connecting all the districts of the body: epidermis, dermis, fat, blood, lymph, blood and lymphatic vessels, tissue covering the nervous filaments (endoneurium, perineurium, epineurium), voluntary striated muscle fibers and the tissue covering and permeating it (epimysium, perimysium, endomysium), ligaments, tendons, aponeurosis, cartilage, bones, meninges, involuntary striated musculature and involuntary smooth muscle (all viscera derived from the mesoderm), visceral ligaments, epiploon (small and large), peritoneum, and tongue. The continuum constantly transmits and receives mechano-metabolic information that can influence the shape and function of the entire body. These afferent/efferent impulses come from the fascia and the tissues that are not considered as part of the fascia in a bi-univocal mode” (Figure 1) [32].

Fascial continuum embryology

During gastrulation, the axial (notochord), paraxial (somites), and lateral areas derive from the mesoderm; the latter will further divide (in the post-gastrulation phase) into anterior and posterior domains [45]. The mesoderm shares several transcriptional pathways with the ectoderm, in particular with the neural crests and ectomesenchyme (ectoderm-derived mesenchyme); neural crest cells migrate (delaminate) from the dorsal neural tube to several different organs and areas of the body [46,47]. From these two layers, mesoderm and ectoderm, in a perfect ontogenetic union will derive the tissues that will constitute the fascial continuum (solid and fluid fascia), which falls within the definition of FORCE. Other organizations that try to define fascial tissue as "connective" do not specify the embryological origin of the same tissue, which has a double phylogeny, contradicting some scientific notions. If only the connective tissue is considered fascia, and since the same connective tissue also derives from the ectoderm, how to consider the other tissues that derive from the mesoderm and the ectoderm? And how to consider the part of the connective tissue that derives from the ectoderm itself? FORCE remedies these gaps by defining the previous paragraph.

Taking some examples, the meninges of the skull, which are considered a fascia by other organizations and ours, are organized in three layers (albeit interpenetrated); the dura mater of the caudal midbrain and forebrain area, the dura mater of the cerebellar tentorium and the large cerebral falx derives from the ectoderm, while the dura mater of the remaining cerebral area has a mesodermal derivation [36]. The tentorium cerebelli, a junction area of biomechanical, biochemical, and fluidic information, consists of an outer dural layer of mesodermal derivation, while the pia and arachnoid have an ectodermal origin [48]. The question of what to introduce in the classical definitions to delimit the concept of fascia arises from the double phylogenetic origin of the craniocervical myofascial area. The 60 contractile districts of the skull formed by precursor cells of muscle fibers (myoblasts) arise from the mesoderm, but the connective tissue that separates the various components and allows them to fuse with the bone tissue derives from the ectoderm (including the tongue) [36]. The connective tissue that creates the shape of the districts of the trapezius muscle area, and of the sternocleidomastoid muscle, derives from both the mesodermal and ectodermal layers [36]. Likewise, the bone tissue of the cranial area has a double phylogeny; some will derive directly from the mesoderm (parietal bone), other bone portions from the ecotoderm (maxillary bone), while still others derive from a fusion of both embryological sheets (frontal bone) [36]. Some bone sutures, such as those that delimit the border between the two parietal bones, have a suture that derives from the ectoderm [36]. If the same human body tissue has different embryological origins, how are its boundaries accurately delineated by the classical definitions? In the definition of FORCE, this question does not arise.

Furthermore, several bone progenitor cells will have a non-bone formation fate (neural tube and dura mater), and marking a precise boundary between the ectoderm and the mesoderm in humans creates an error in the final considerations of the classic interpretations and definitions of what is the fascial tissue [36]. Other tissues are classified as specialized connective tissue in medical texts, yet they are not in the least considered as fascial tissue by most authors (blood, lymph, cephalorachid fluid, and bones) [49,50]. This is another contradiction. Why? Probably, those who started using the terminology of fascia for manual medicine did not know how to use manual techniques for such tissues and, now, it is too late to go back [51-53]. "The difficulty lies not so much in developing new ideas as in escaping from old ones" [4].

The importance of the fluid fascia

Body fluids (fluid fascia) give shape and function to the body (solid fascia) [42]. The lymphatic and blood vessels cross the entire body, from the epidermis to the bone, from the viscera to the nervous system (as well as the neural ramifications). The fluid network (FN) and the neural network (NN) pervade the whole body, and despite the clamor of complex innervation on solid fascial tissue (for some authors only what it envelops), if there were no FN/NN, there would not even be the function, the form, and the eventual symptoms [54-57]. Before the movement, the pathways of nourishment, cleansing, and information must be created. The blood vessels are sensitive not only to external pressure but to the different modes of passage of fluids; as fluids behave, so will the tissues that transport them (vessels) and the tissues they pass through (from bones to skin) adapt, determining form and function [58]. Erythrocytes and macrophages, cells of the blood and lymphatic system, change shape and function according to the pressures they feel during their transport by fluids (thanks to PIEZO1, a mechanotransducer or ion channel membrane protein) [59]. Erythrocytes influence the vasodilation/vasoconstriction mechanism of the blood vessel, through a complex relationship between the nitric oxide produced by the erythrocyte membrane and the sodium-potassium protein channels of the smooth muscle membrane [60]. Macrophages carry out inflammatory responses when the deformation of their membrane exceeds a certain threshold of mechanical signal (therefore, not only for chemical signals), due to the pressures of the fluid with which they are transported [61-62].

Interstitial fluids-extracellular matrix (IFEM)

A fluidic component of the human body is the IFEM; this represents about 40% of body mass and contains about 30% of body proteins [63]. The IFEM is able to influence the shape and function of cells and tissues. To give an example, the movement of these fluids in the bones in the lacunar-canaliculi network allows osteocytes to perceive and broaden the perception to mechanical stress, improving the mechanotransductive response [64]. The fluids between the osteocytes allow the same bone cells to communicate with each other, in response to the directional and pressure patterns of the fluids, thanks to the biochemical elements transported, and due to their ubiquity, any change in fluid displacement is immediately felt by the osteocytic cells [64]. The osteogenic process has a greater impact not thanks to the direct mechanical deformation of the osteocyte membrane (the support of the foot on the ground), but thanks to the flow of fluids between the cells [64]. The IFEM works as a non-neural network, but with the same goal, that is, to communicate and receive. The way fluids move affects the behavior of immune cells. Thanks to certain fluid movement patterns (speed, quantity, viscosity, turbulence, direction), the leukocyte recognizes the immune behavior to be pursued (adhesion, migration, activation), thanks to membrane proteins (selectins) activated by fluidic stimuli; the mechanical stimulus deriving from fluids is crucial for a correct immune response [65]. The IFEM possesses viscoelastic properties, thanks to components such as collagen, elastic fibers (elastin, fibrillin), glycosaminoglycans, water, and polysaccharides; without these fluids, cells, and tissues would not be able to communicate properly and would not slide/move [66]. Lack of movement and adequate communication leads to disease, pain, and inflammation [66]. The IFEM is found throughout the body, connecting the whole body, regardless of the layers or anatomical areas; it creates a continuity where all structures, local or systemic, are in contact, with a volume of fluids that is three times more than the sum of the blood and lymphatic volume [67]. The IFEM is constantly changing and represents another fluid circulation system [67]. The transport of fluids occurs due to the movement of muscles and the viscera and respiration [67]. In particular, the blood vessels (venous and arterial) transport interstitial fluids in a double way. A part of the IFEM passes between the tunica adventitia and the paravascular layer, with a speed of about 0.1-7.6 millimeters per second; the latter is a set of loose connective tissue that makes the blood vessel stable with respect to the surrounding tissues [68]. The IFEM transported to the heart between the vessel and the paravascular area has a longitudinal flow. There is another transverse direction of flow, through pores within the tunica adventitia with a speed of about 3.6-15.6 millimeters per second; the fluids pass between the fibers that make up the tunic [68]. These mechanisms of different directions probably serve to filter fluids (different molecular sizes); the same filtering mechanism serves to correctly transport biomolecular signals to distant areas and electrical charges [68].

We find a similar mechanism in the glymphatic system [68,69]. The cerebrospinal fluid (CSF), which exchanges information with the glymphatic and venous system, according to recent research, communicates with some medullary receptors within the bones of the skull to modulate a possible neuroinflammatory response [70]. It travels between the perivascular space of the dural vessels (from the subarachnoid space), to reach the bone marrow of the bones of the skull, through bone channels; moreover, this journey is bidirectional, that is, from the dura to the bone marrow and vice versa [70]. The cranial bone marrow discriminates the quality of the CSF composition and, based on the substances transported, it could send biochemical signals (inflammatory or non-inflammatory) toward the nervous system [70]. The IFEM transports not only chemical molecules but also cells. Cells have their own bioelectric field, which can become a communication tool to other cells, altering their electric field [71]. The fluid composition of the IFEM carries other types of messages, such as electromagnetic currents, which can involve areas distant from the physical passage of fluids [68]. This electrical and magnetic information allows cells near and far to receive the same information, although the same cells may have different responses; this mechanism allows the tissues to respect their morphogenetic behavior or morphic field [71]. The constant movement of fluids or fluid fascia ensures systemic electromagnetic integration and cellular cohesion [72]. The fluids carry the electromagnetic fields of cellular DNA, in order to maintain tissue memory and share this memory with all tissues [72]. The heartbeat itself generates electromagnetic fields, which are transported and distributed to various tissues by fluids [73].

From the theoretical model of tensegrity to that of fascintegrity

**Figure 2:**
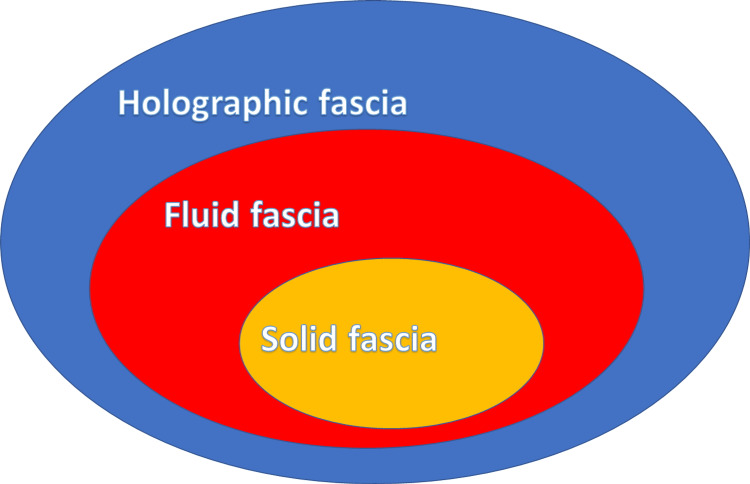
Schematic diagram illustrating the greater influence of the oscillations (holographic fascia), compared to the fluid fascia and the solid fascia. It is the nano-movements of biophotons and biophonons that determine behavior at the macroscopic level Figure Source: Bruno Bordoni

The term "tensegrity" (tensional integrity) derives from an architectural concept, conceived by the designer R. Buckminster Fuller in 1960; a solid structure capable of managing tension variations, through structures capable of absorbing and transmitting mechanical tension (continuous tension with discontinuous compression) [57]. Dr. Robbie (1977) transported the concept of tensegrity into the field of biology by attempting to determine the mechanical behavior between the spinal column and muscle structure acting on the vertebrae [57]. Dr. Ingber, in the 1970s, took a further step, that is, he tried to describe the behavior of the cell, always from a mechanical point of view, with the concept of tensegrity, where the microtubules represent the continuous tension and the actomyosin protein complex represents the discontinuous compression [57]. Dr. Levin, in 1981, presented a poster at the 34th Annual Conference on Engineering in Medicine and Biology, where he inserted the term biotensegrity, combining the architectural concept with a purely biological field; this theory considers the bone tissue as the component in discontinuous mechanical tension, while the muscles and joints represent the component in constant tension or in pre-stress (Figure 2) [57]. Figure 2 illustrates the classic view of the human body formed solely by solid fascial tissue (in this case, muscles), forgetting the concept of the fluid fascia.

In 2022, the term biotensegrity is still used to explain biological mechanical behavior, from cell to tissue, but without considering the fluids and other informational transport mechanisms that are able to influence cellular behavior, the theoretical model loses its value [57]. In 2019, our research group (FORCE) coined a new term, to try to conceptualize the behavior of the living, that is, "fascintegrity"; the word combines the term of tensegrity with the concept of fascial continuum (solid and fluid) [74]. Recall that, when a theoretical model is not proven by experimental studies, the model remains theoretical; the mere fact of reporting the term and the concept it expresses a multitude of times does not make this model magically valid [75,76]. The model of biotensegrity and fascintegrity remain, for now, only conceptual theories. What makes fascintegrity more relevant is the inclusion of fluids (blood, lymph, extracellular matrix, and interstitial fluids) in the fascial concept. In this update article, we wish to add another little-considered aspect in understanding cellular and tissue behavior: other communication tools in the living system, which do not fit into the biotensecretive model.

Oscillations

The oscillations or vibrations, that is, electromagnetic frequencies, concern the whole universe; a communication system of which we are an integral part, as emitters and receivers [77]. Any form of force that alters the shape of the cell is followed by mechanotransduction, with pleiotropic effects; this force can be mechanical or electrical energies, electromagnetic fields, and radiations (light and sound) [77]. Each force has a code, highlighted in the form of wavelength, frequency, direction, types of molecules, and more. The principle of each cell, DNA, is an oscillatory structure capable of resonating in response to other electromagnetic frequencies; these oscillations move the electrons of the DNA, allowing the different proteins that compose it to act so as to remodel or stabilize the double helices [77]. DNA has and recognizes specific spectral signatures in order to create local and distant connections [77]. The specific response to DNA oscillations is then handled by the cell's microtubules and microfilaments, just like a chip or a micro-brain [77-79]. This view of biological behavior is based on quantum biology, where we move from the microscale to the nanoscale [79]. Each cell has memory and awareness, regardless of neural presence [79]. Compared to the concept of biotensegrity, where the cell or tissue has no awareness or initiative, quantum biology allows the concept of fascial continuum to evolve, where the model of fascintegrity becomes dynamic and active. The cell (and therefore, all tissues at the macroscopic level) collects information (senes); the sum of the information encoded by the DNA is the senome [80]. The senome responds and adapts constantly with electrical and then molecular activity [80]. The senome does not need matter to evolve, as it derives from energy, such as light and sound (oscillations or fields of electromagnetic energy); through matter (from DNA and towards all tissues and vice versa), the senome creates the dynamic informational inter-reciprocity of multiple electromagnetic fields, making the tissues cohesive [80,81].

Holographic fascia

The electromagnetic field interacts through particles such as biophotons (light) and biophonons (sound), which create an instrument of dialogue with matter through electrical charges [81]. All cells throughout the body are in communication through the vision of quantum biology [82]. When the DNA and all the components of the cell activate specific responses to biophotons and biophonons (which arrive with oscillations), the electrons of the double helices and the different cellular structures respond, creating oscillations of equal amplitude and rhythm, emitting new biophotons and phonons; these expand, creating new electromagnetic fields, both locally and distally [82]. The human body constantly receives and emits electromagnetic fields to maintain form and function [82-84]. The electromagnetic information travels as flows [85]. We could talk about the holographic fascia. The biophonons are generated by the cell when the same is altered in its shape (nano-movements), by the oscillations of the biophotons; light creates responses of cellular structures, including biophonons [86]. Light and sound arrive and derive from the cells, allowing a systemic dialogue [86]. We could say that the human being is the response to a harmonic coherence of light and sound. The same myofascia (muscular complex) when it performs an action, produces a sound, which can be recorded by sensitive equipment (from the stethoscope to a microphone on the skin), with a frequency of 20-30Hz [87]. According to the hypothesis of quantum physics, there are quantum fields (light and sound) that influence the perception of matter by our senses, creating subjectivity. Quantum physics itself teaches us that we can interact with such fields and form our own quantum fields. On the one hand, we are influenced by what we perceive with touch, but on the other hand, we can change the matter we touch, making osteopathic medicine very concrete [88-89]. A concept similar to quantum biology is described in 1981 by Rupert Sheldrake, with morphic fields. The morphic (or morphogenetic) field is an information field, a field of consciousness that contains all the information relating to a specific species. It is a field to which everyone is connected and with which everyone enters into a relationship (morphic resonance); it is a kind of collective consciousness, a single consciousness made up of the consciousness of all individuals. This means that by increasing one's awareness, it also increases the collective consciousness, but also vice versa, that is, the more the collective consciousness increases, the more one's consciousness will increase as a result of resonance. [90]. We feel everything and become in everything. Feeling is already movement and transformation. It is not possible for the living to separate from the whole, because we are everything. In histology, many cells are found ubiquitously in different tissues, such as fibroblasts and telocytes: how to consider this lack of functional tissue demarcation in a fascial view? Another problem for the conventional view of the fascial system, which recalls the uniqueness of quantum biology. The concept of biotensegrity has no practical value for understanding the actions that occur at the level of biological activity, which can be measured on a nanoscale; it is the nano-movements of biophotons and biophonons that determine behavior at the macroscopic level [91-96].

The fascial continuum today

The complexity of the fascial continuum is not yet fully understood. It is a mistake to enclose the fascia in a mere anatomical and histological vision because other sciences are telling us that the approach to the fascial system must change in order to achieve greater clinical incisiveness. Mechanistic-metabolic thinking alone is not enough, although it is an excellent principle. Compared to the previous update of 2021, and by adding new information reported in the article, we have made small changes to the definition that FORCE supports on the fascial continuum; the changes are indicated as follows:

“the fascia is any tissue that contains features capable of responding to mechanical stimuli. The fascial continuum is the result of the evolution of the perfect synergy among different tissues, fluids, and solids, capable of supporting, dividing, penetrating, feeding, and connecting all the districts of the body: epidermis, dermis, fat, blood, lymph, blood and lymphatic vessels, tissue covering the nervous filaments (endoneurium, perineurium, epineurium and circumneurium), voluntary striated muscle fibers and the tissue covering and permeating it (epimysium, perimysium, endomysium), ligaments, tendons, aponeurosis, cartilage, bones, meninges, involuntary striated musculature and involuntary smooth muscle (all viscera derived from the mesoderm), visceral ligaments, epiploon (small and large), peritoneum, and tongue. The continuum constantly transmits and receives mechano-metabolic-quantum information that can influence the shape and function of the entire body. These afferent/efferent information come from the fascia and the tissues that are not considered as part of the fascia in a bi-univocal mode.”

**Figure 3:**
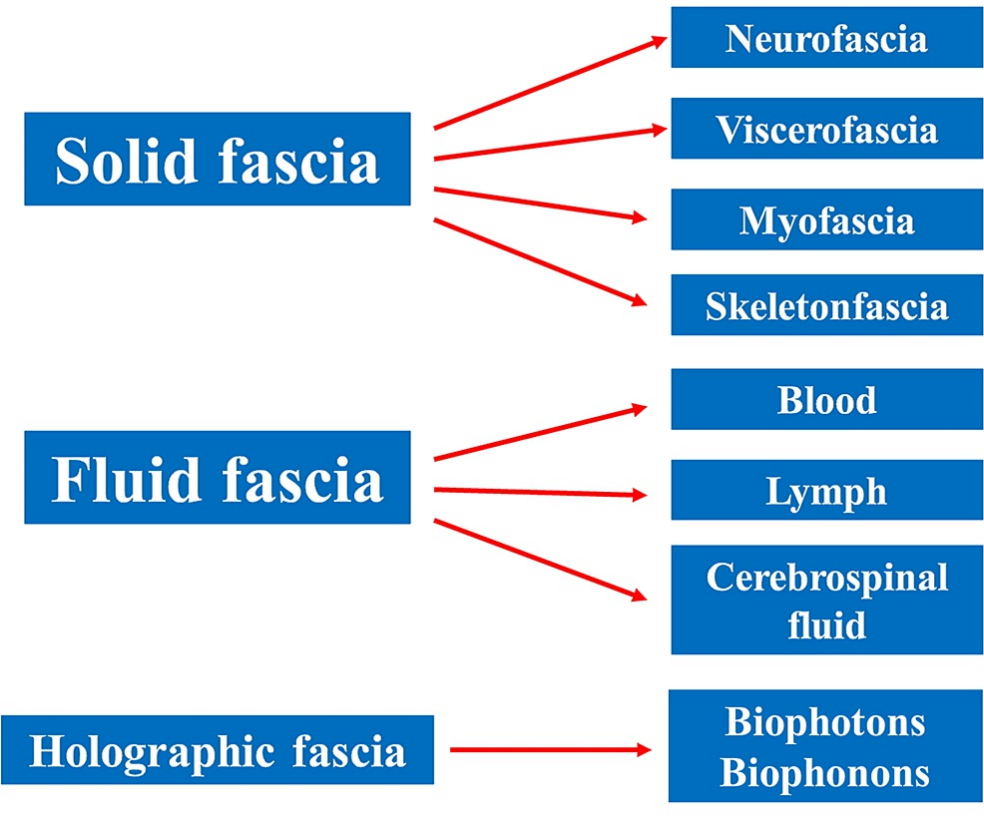
The subdivision highlights the existence of the holographic fascia, compared to the classic fascial subdivisions; in addition, the list recalls the existence of different tissues, such as bone and fluid fascia Figure Source: Bruno Bordoni

Evolution requires us to observe the same matter with different thoughts [97]. Figures 2, 3 summarize some concepts of the article. Figure 2 highlights the importance of the oscillations with respect to the more usual optics in observing the fascia. 

Figure 3 schematizes the subdivision of the solid and fluid fascia, with the addition of the holographic fascia, trying to bring innovation to the understanding of the fascial continuum. 

Matter is constantly evolving and each energy signature (the specific way in which a structure deforms) is a communicative interface; the fascia is not a set of cells, but a set of different energy morphologies [97].

## Conclusions

When trying to deal with the understanding of the fascial continuum, it is necessary to keep in mind the different scientific disciplines that constitute and study the human body, and not just some subjects for the convenience of pedagogy. The article included components of embryology and quantum biology, subjects that are rarely included to frame the concept of fascia. The fascia has solid, fluid, and electromagnetic components, which create a perfect functional mosaic observable at the macroscopic and nanoscopic level. The article reviewed the concept and information that FORCE has been offering for several years on the fascial continuum, a non-profit organization with no copyright and includes many scientific figures from different backgrounds. Fascial tissue is concerned with the patient's health, and should be viewed as an important tool for finding more suitable solutions for maintaining the same health. Understanding and applying are not always synonymous. We hope that the study of this wonderful biological field will evolve more and more, without authoritarianism or economic interests.
